# De-escalated rivaroxaban in postoperative isolated distal deep vein thrombosis: a propensity score–matched study

**DOI:** 10.3389/fcvm.2026.1855986

**Published:** 2026-10-19

**Authors:** Hansheng Bai, Qingzhu Tang, Linhao Jin, Mengqi Yang, Guangjun Fan, Si Wu

**Affiliations:** 1 Department of Pharmacy, The Second Affiliated Hospital of Dalian Medical University, Dalian, China; 2 Department of Nephrology, The First Affiliated Hospital of Dalian Medical University, Dalian, China; 3 College of Pharmacy, Dalian Medical University, Dalian, China

**Keywords:** anticoagulation intensity, bleeding risk, direct oral anticoagulants (DOACs), isolated distal deep vein thrombosis, postoperative complications

## Abstract

**Background:**

In postoperative isolated distal deep vein thrombosis (IDDVT), anticoagulation intensity is frequently constrained by bleeding risk, which may compromise treatment continuity and clinical effectiveness.

**Objectives:**

To compare the effectiveness and safety of a de-escalated (rivaroxaban 10 mg once daily) versus standard-maintenance rivaroxaban regimen for postoperative IDDVT in a real-world surgical cohort.

**Methods:**

This single-center retrospective cohort study included patients diagnosed with IDDVT following urological surgery between 2013 and 2025. Patients received rivaroxaban at either a low maintenance dose (10 mg once daily) or a standard maintenance dose (20 mg once daily) according to routine clinical practice. Propensity score matching (1:1) was applied to balance baseline characteristics, incorporating surgical approach (endoscopic vs. major structural procedures) as a surrogate for surgical trauma and bleeding risk. The primary outcome was thrombus progression to proximal deep vein thrombosis or pulmonary embolism. Secondary outcomes included IDDVT resolution and clinically relevant non-major bleeding (CRNMB). Competing-risk analyses were performed.

**Results:**

After matching, 152 patients were included (76 per group). Thrombus progression did not differ significantly between groups (subdistribution hazard ratio [SHR] 1.29; 95% CI 0.48–3.46; *P* = 0.613). No statistically significant difference in IDDVT resolution was detected between the two groups (SHR 0.93; 95% CI, 0.64–1.36; *P* = 0.721). The low-dose regimen was associated with a significantly lower risk of CRNMB (HR 0.31; 95% CI, 0.11–0.85; *P* = 0.023) and fewer early bleeding-related treatment interruptions. Early postoperative hematuria was more frequent in the standard-dose group.

**Conclusions:**

In this single-center retrospective cohort of patients with isolated distal deep vein thrombosis after urological surgery, low-dose rivaroxaban (10 mg once daily) was associated with a lower incidence of clinically relevant non-major bleeding than 20 mg once daily. No statistically significant differences were detected between regimens in thrombus progression or complete thrombus resolution. However, these findings should not be interpreted as evidence of equivalent antithrombotic efficacy, given nonrandomized treatment allocation, residual confounding, limited sample size, and wide confidence intervals for thrombotic outcomes. The results are hypothesis-generating and support studies—preferably randomized non-inferiority trials—to determine whether dose de-escalation can provide an acceptable balance between thrombotic protection and bleeding risk in postoperative patients.

## 1 Introduction

Venous thromboembolism (VTE) is a major cause of perioperative morbidity and mortality, contributing to prolonged hospitalization, increased readmission rates, and long-term functional impairment (1, 2). Current clinical guidelines emphasize individualized risk assessment and prevention strategies (3). However, in surgical settings where thrombotic and bleeding risks frequently coexist—particularly following abdominopelvic procedures involving hypervascular organs—the optimal intensity and timing of anticoagulation remain uncertain.

Isolated distal deep vein thrombosis (IDDVT), defined as thrombosis confined to the infrapopliteal deep veins without proximal extension or pulmonary involvement, has historically been regarded as a relatively benign condition. Emerging evidence, however, indicates a heterogeneous clinical course, with risks of proximal propagation, recurrence, and pulmonary embolism, particularly among patients receiving no anticoagulation or inadequate treatment (4, 5). Compared with proximal DVT, IDDVT is more commonly associated with transient provoking factors such as recent surgery or trauma, while chronic comorbidity burden is often lower, suggesting that evidence derived predominantly from non-surgical populations may not be fully generalizable (6).

Most contemporary guidelines recommend at least three months of anticoagulation for patients with high-risk IDDVT, with direct oral anticoagulants preferred when feasible (1, 3). These recommendations, however, are largely extrapolated from randomized trials enrolling non-surgical or mixed populations, as patients at high risk of postoperative bleeding are frequently excluded (7). Consequently, uncertainty persists regarding the balance between thrombotic prevention and bleeding risk in postoperative patients, and more conservative anticoagulation strategies are often observed in real-world practice (8, 9).

Randomized trials in the broader VTE population have demonstrated that extended treatment with low-dose rivaroxaban maintains antithrombotic efficacy (7). Whether this strategy can be applied to patients with IDDVT in postoperative settings remains unclear, as most available studies have focused on standard-dose regimens. To address this gap, we conducted a propensity score–matched analysis of a real-world perioperative cohort to compare the effectiveness and safety of low- versus standard-maintenance dose rivaroxaban in patients diagnosed with IDDVT following urological surgery, focusing on thrombus progression, thrombus resolution, and bleeding outcomes.

## 2 Methods

### 2.1 Study design and patient population

This was a single-center, retrospective cohort study conducted at the Second Hospital of Dalian Medical University. Consecutive patients who underwent urological surgery between January 2013 and April 2025 and were diagnosed with IDDVT by postoperative lower-extremity duplex ultrasonography were screened for inclusion. Urological procedures included robot-assisted or laparoscopic radical prostatectomy, radical or partial nephrectomy, transurethral resection of the prostate, transurethral resection of bladder tumors, cystoscopy, and other related procedures. This study was approved by the institutional review board of The Second Hospital of Dalian Medical University. The requirement for informed consent was waived due to the retrospective nature of the study.

Eligible patients were hospitalized adults who met the following inclusion criteria: (1) urological surgery performed at our institution between 2013 and 2025; (2) postoperative ultrasonographic confirmation of IDDVT; (3) normal preoperative coagulation function; (4) no contraindications to anticoagulation; and (5) complete clinical data.

Patients were excluded if they met any of the following criteria: (1) renal insufficiency (creatinine clearance <50 mL/min); (2) moderate to severe hepatic dysfunction; (3) active bleeding prior to initiation of anticoagulation; (4) congenital or acquired coagulation disorders; (5) preoperative proximal deep vein thrombosis (PDVT) or pulmonary embolism (PTE); (6) regular anticoagulant or antiplatelet therapy prior to surgery; or (7) loss to follow-up.

All patients diagnosed with IDDVT received anticoagulation therapy after exclusion of contraindications. According to the postoperative rivaroxaban maintenance dose administered in routine clinical practice, patients were categorized into two groups: a standard maintenance dose (SMD) group receiving rivaroxaban 20 mg once daily, and a low maintenance dose (LMD) group receiving rivaroxaban 10 mg once daily (the de-escalated regimen). Anticoagulation therapy was prescribed for 3 months and extended to 6 months if thrombus resolution was not observed, at the discretion of the treating physician. Because the study period spanned more than a decade, year of surgery was incorporated into the propensity score model to account for potential temporal changes in surgical practice, anticoagulation management, and patient selection. In routine clinical practice, the rivaroxaban dose was determined by the treating physicians rather than assigned according to a prespecified dosing protocol. Dose selection was based on the physicians’ overall assessment of patient-specific factors, including advanced age, surgical complexity, the anticipated risk of bleeding events, and other relevant clinical considerations. However, standardized documentation and explicit dose-selection criteria were not available in the medical records; therefore, the independent influence of physician preference on dose selection could not be reliably determined.

Follow-up duplex ultrasonography was generally recommended at approximately 1 month after IDDVT diagnosis. If complete thrombus resolution was not documented, repeat ultrasonography was performed at approximately 3 months and, if the thrombus remained unresolved, follow-up was extended to approximately 6 months. Because this was a retrospective study without a prespecified imaging schedule, the actual timing of ultrasonographic examinations varied according to patients’ follow-up attendance and clinical visit schedules

### 2.2 Data collection

Clinical data were collected from the electronic medical record system, including demographic characteristics (age, sex, and body mass index), medical history and comorbidities (hypertension, diabetes mellitus, mild renal dysfunction, mild hepatic dysfunction, malignancy, and prior chemo-radiotherapy), perioperative information, laboratory parameters (including D-dimer levels and platelet counts), and imaging findings (location of IDDVT, timing of diagnosis, and thrombus outcomes during follow-up). All outcome events were confirmed through postoperative follow-up at 3 and 6 months, including repeat duplex ultrasonography.

### 2.3 Outcome definitions

The primary outcome was thrombus progression to proximal deep vein thrombosis (PDVT) or the occurrence of pulmonary embolism (PTE). Secondary outcomes included complete thrombus resolution, defined as complete disappearance of thrombus on follow-up ultrasonography, and bleeding events.

Bleeding events were classified according to the criteria of the International Society on Thrombosis and Haemostasis (ISTH), including major bleeding and clinically relevant non-major bleeding (CRNMB). Hematuria was recorded and analyzed as a separate bleeding manifestation.

### 2.4 Statistical analysis

In the unmatched cohort, continuous variables are presented as mean ± standard deviation and were compared using the independent samples t-test or the Mann–Whitney U test, as appropriate. Categorical variables are presented as counts (percentages) and were compared using the chi-square test or Fisher's exact test. To account for variations in baseline surgical trauma and expected bleeding patterns, the procedures were dichotomized based on the surgical approach: endoscopic procedures (e.g., transurethral resections, utilizing natural orifices with localized bleeding risks) versus major structural surgeries (including robotic-assisted, laparoscopic, and open procedures, which involve abdominal/retroperitoneal dissection and carry higher systemic bleeding and prothrombotic risks).

To reduce bias from measurable confounding factors between groups, a propensity score matching (PSM) model was constructed based on age, sex, body mass index, platelet count, D-dimer level, hypertension, diabetes mellitus, mild renal dysfunction, mild hepatic dysfunction, surgical approach (endoscopic procedure versus laparoscopic/open surgery), NSAID use, perioperative immobilization, malignancy, history of acute myocardial infarction, congestive heart failure, stroke history, chemoradiotherapy history, year of surgery (2013–2016, 2017–2020, or 2021–2024) and history of venous thromboembolism. Patients were matched 1:1 using the nearest-neighbor method to generate the matched cohort. When the absolute mean standardized difference was under 0.2, it was considered balanced matching (10). For outcome analyses, thrombus progression and complete thrombus resolution were evaluated using Fine–Gray competing risk models, with treatment discontinuation due to bleeding treated as a competing event. Subdistribution hazard ratios (SHRs) and corresponding 95% confidence intervals (CIs) were reported. Bleeding events were analyzed using Cox proportional hazards models, and hazard ratios (HRs) with 95% CIs were calculated. Cumulative Incidence Functions (CIFs) were compared using Gray's test (for thrombus outcomes) and the survival curves were compared using the Log-rank test (for bleeding events).

All statistical analyses were performed using Stata version 17 (Stata Corp, College Station, TX, USA) and SPSS version 22 (IBM Corp, Armonk, NY, USA). *P* values were reported to three decimal places, and a two-sided *P* value <0.05 was considered statistically significant.

## 3 Results

Between January 2013 and April 2025, 302 patients diagnosed with perioperative IDDVT via ultrasound were screened. Fifty-five patients were excluded due to incomplete follow-up, concurrent PDVT or PE, significant admission bleeding, or other indications for anticoagulation. No patients from 2025 met the eligibility criteria and were included in the initial cohort. Consequently, 247 patients were included in the initial cohort (Figure 1). Following 1:1 PSM, 152 patients (76 per group) were retained for the comparative analysis. Baseline characteristics are summarized in Table 1. In the matched cohort, the earliest surgery dates were January 2014 in the SMD group and May 2013 in the LMD group. The distribution of surgery periods was well balanced between the two groups after propensity score matching (standardized difference = 0.039). All variables listed in the table were included in the propensity score matching analysis.

**Figure 1:**
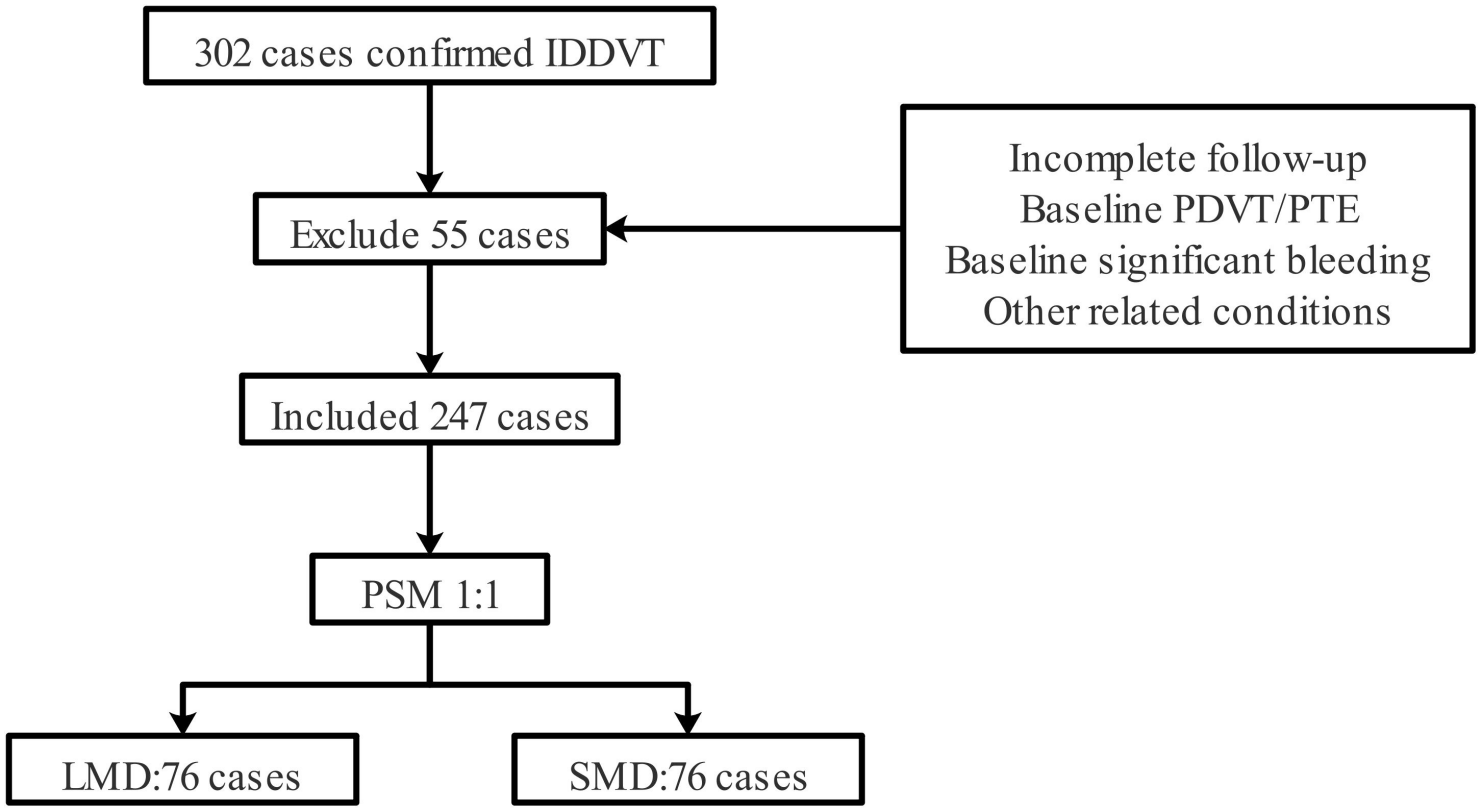
Participant inclusion and matching process.

**Table 1:** Baseline characteristics in unmatched and matched cohorts.

Characteristics	Unmatched				PSM			
	SMD *n* = 116	LMD *n* = 131	*P*	Std. Diff.	SMD *n* = 76	LMD *n* = 76	*P*	Std. Diff.
Age(years), MN (SD)	72.72 (10.86)	72.55 (10.77)	0.899	−0.016	72.13 (11.98)	72.01 (11.71)	0.870	−0.010
BMI(kg/m^2^), MN (SD)	23.97 (3.93)	24.07 (3.53)	0.844	0.025	23.81 (3.87)	24.01 (3.75)	0.924	0.052
PLT, 10^9^/L, MN (SD)	200.96 (82.14)	189.59 (78.57)	0.268	−0.141	192.23 (82.96)	203.37 (84.30)	0.275	0.133
D-dimer (mg/L), MN (SD)	1.00 (0.57)	1.02 (0.60)	0.748	0.041	1.00 (0.56)	1.03 (0.59)	0.828	0.057
Male, *n* (%)	81 (69.83)	98 (74.81)	0.383	0.111	57 (75.00)	58 (76.32)	0.850	0.031
HTN, *n* (%)	51 (43.97)	55 (41.98)	0.754	−0.040	31 (40.79)	32 (42.11)	0.869	0.027
DM, *n* (%)	22 (18.97)	25 (19.08)	0.981	0.003	15 (19.74)	17 (22.37)	0.691	0.065
RI, *n* (%)	11 (9.48)	9 (6.87)	0.453	−0.095	6 (7.89)	7 (9.21)	0.772	0.047
HI, *n* (%)	4 (3.45)	6 (4.58)	0.754	0.058	2 (2.63)	2 (2.63)	1.000	0.000
Surgical Approach, *n* (%)			<0.001	0.545			0.741	−0.054
- Endoscopic procedure	54 (46.55)	96 (73.28)			46 (60.53)	44 (57.89)		
- Laparoscopic/Open	62 (53.45)	35 (26.72)			30 (39.47)	32 (42.11)		
NSAIDs, *n* (%)	67 (57.76)	81 (61.83)	0.515	0.083	47 (61.84)	46 (60.53)	0.868	−0.027
Bedridden(≥3days), *n* (%)	6 (5.17)	6 (4.58)	0.829	−0.027	3 (3.95)	3 (3.95)	1.000	0.000
Malignancy history, *n* (%)	63 (54.31)	81 (61.83)	0.232	0.152	41 (53.95)	44 (57.89)	0.624	0.080
AMI history, *n* (%)	4 (3.45)	9 (6.87)	0.230	0.155	4 (5.26)	4 (5.26)	1.000	0.000
CHF history, *n* (%)	4 (3.45)	3 (2.29)	0.709	−0.069	3 (3.95)	2 (2.63)	1.000	−0.074
Stroke history, *n* (%)	14 (12.07)	13 (9.92)	0.59	−0.069	7 (9.21)	8 (10.53)	0.786	0.044
Chemo-radiotherapy, *n* (%)	2 (1.72)	5 (3.82)	0.453	0.128	2 (2.63)	3 (3.95)	1.000	0.074
Year of surgery, *n* (%)			0.005	0.412			0.809	0.039
2013–2016	24 (20.69)	15 (11.45)			13 (17.11)	12 (15.79)		
2017–2020	48 (41.38)	40 (30.53)			27 (35.53)	27 (35.53)		
2021–2024	44 (37.93)	76 (57.82)			36 (47.37)	37 (48.68)		
VTE history, *n* (%)	19 (16.38)	15 (11.45)	0.263	−0.142	12 (15.79)	12 (15.79)	0.645	−0.075

Data are presented as mean (SD) or *n* (%). Continuous variables were compared using the Student's t test or Mann–Whitney U test, and categorical variables using the *χ*^2^ test or Fisher's exact test, as appropriate. Propensity score matching (1:1) was applied. A two-sided *P* < 0.05 was considered statistically significant. Balanced matching was defined as an absolute mean standardized difference < 0.2. SMD, standard maintenance-dose anticoagulation; LMD, low maintenance-dose anticoagulation; Std. Diff, standardized mean difference; BMI, body mass index; PLT, platelet count; DM, diabetes mellitus; HTN, hypertension; RI, renal insufficiency; HI, hepatic insufficiency; NSAIDs, nonsteroidal anti-inflammatory drugs; AMI, acute myocardial infarction; CHF, congestive heart failure; VTE, venous thromboembolism.

The timing and course of rivaroxaban anticoagulation are summarized in Table 2. The median time from surgery to IDDVT diagnosis was 1 day (IQR, 1–2) in the SMD group and 2 days (IQR, 1–3) in the LMD group, while the median time from IDDVT diagnosis to rivaroxaban initiation was 3 days (IQR, 1–6) and 3 days (IQR, 2–5), respectively. Anticoagulation was completed within 3 months in 52 (68.42%) and 59 (77.63%) patients, respectively, and was extended to 6 months in 9 (11.84%) and 12 (15.79%) patients. Bleeding-related treatment interruption occurred in 15 (19.74%) and 5 (6.58%) patients, respectively.

**Table 2:** Timing and course of rivaroxaban anticoagulation.

Variable	SMD (*n* = 76)	LMD (*n* = 76)
Time from surgery to IDDVT diagnosis, days, median (IQR)	1 (1–2)	2 (1–3)
Time from IDDVT diagnosis to rivaroxaban initiation, days, median (IQR)	3 (1–6)	3 (2–5)
Completed within 3 months, *n* (%)	52 (68.42%)	59 (77.63%)
Extension to 6 months, *n* (%)	9 (11.84%)	12 (15.79%)
Bleeding-related treatment interruption, *n* (%)	15 (19.74%)	5 (6.58%)

The categories of anticoagulation course were mutually exclusive and collectively accounted for all patients in each group.

### 3.1 Primary outcome

Regarding the primary outcome (VTE progression), the cumulative incidence at the end of follow-up was 11.84% (95% CI 0.05–0.19) in the LMD group and 9.21% (95% CI 0.02–0.16) in the SMD group. No significant difference was observed between CIFs (Gray's test *P* = 0.256). Consistent with this finding, the Fine–Gray competing risk model yielded an SHR of 1.29 (95% CI 0.48–3.46; *P* = 0.613) for the LMD group compared with the SMD group. The CIFs for both groups are presented in Figure 2.

**Figure 2:**
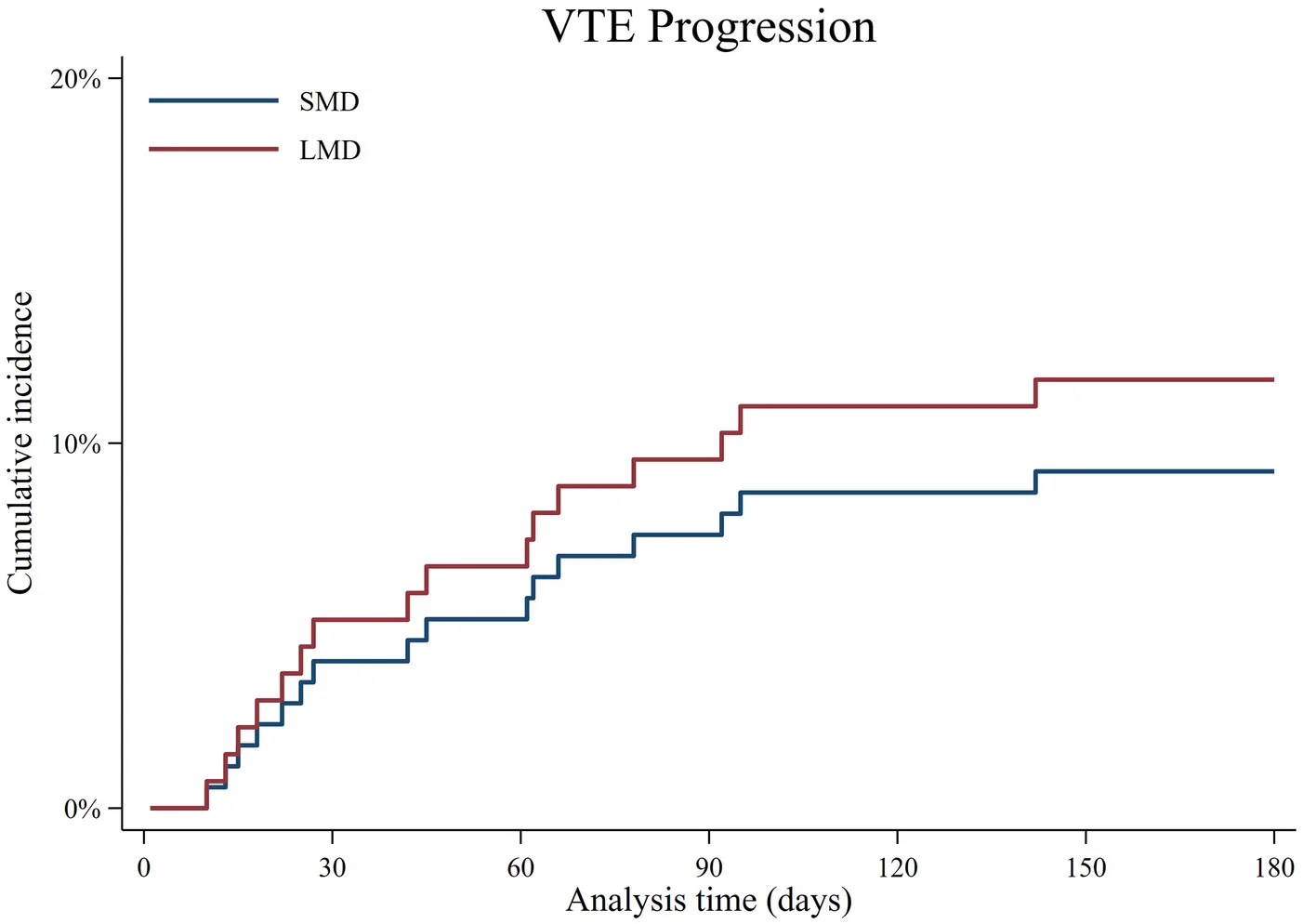
Cumulative incidence of VTE progression. No significant difference was observed (SHR = 1.29; 95% CI, 0.48–3.46; *P* = 0.613).

### 3.2 Secondary outcome

For the secondary outcome of CRNMB, the cumulative incidence at the end of follow-up was 6.58% (95% CI 0.01–0.12) in the LMD group versus 19.74% (95% CI 0.11–0.29) in the SMD group. The Log-rank test demonstrated a statistically significant difference between the cumulative incidence curves (*P* = 0.016). Consistent with this, the Cox proportional hazards model yielded an HR of 0.31 (95% CI 0.11–0.85; *P* = 0.023) for the LMD group. The cumulative incidence curves are presented in Figure 3. The detailed characteristics and management of bleeding events, including bleeding site, time to onset, and subsequent treatment strategy, are summarized in Table 3.

**Figure 3:**
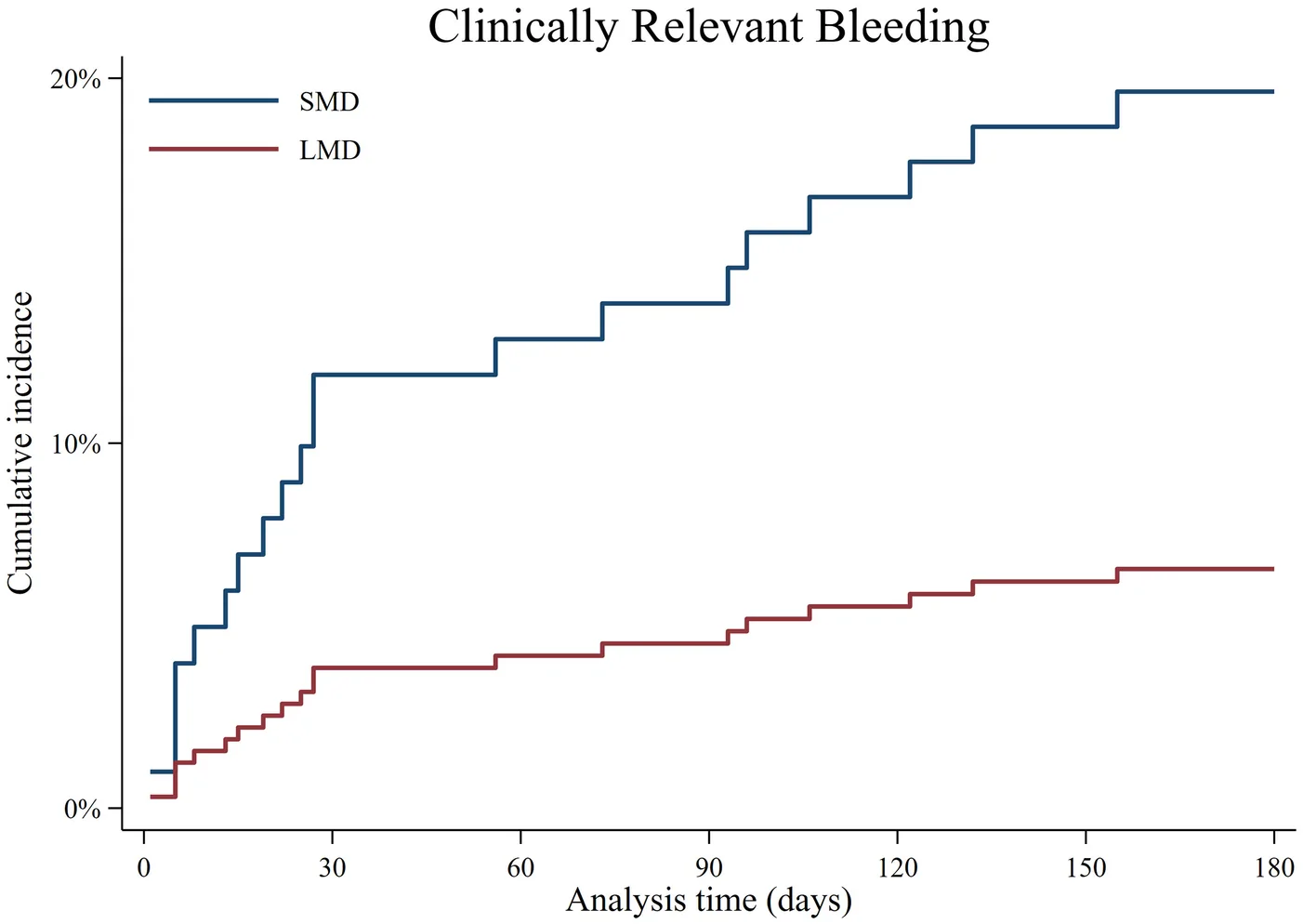
Kaplan–meier curves for clinically relevant non-major bleeding. A significant difference was observed (HR = 0.31; 95% CI, 0.11–0.85; *P* = 0.023).

**Table 3:** Characteristics and management of bleeding events.

Variable	SMD (*n* = 15)	LMD(*n* = 5)
Bleeding site		
Genitourinary system	13 (86.7%)	3 (60.0%)
Gastrointestinal system	0 (0.0%)	2 (40.0%)
Intra-abdominal cavity	2 (13.3%)	0 (0.0%)
Time to bleeding onset, days		
Median (range)	25 (1–155)	27 (8–106)
Post-bleeding management		
Dose reduction	6 (40.0%)	0 (0.0%)
Treatment discontinuation	9 (60.0%)	5 (100.0%)
└─ Discontinuation only	5 (33.3%)	3 (60.0%)
└─ Discontinuation with transfusion	1 (6.7%)	2 (40.0%)
└─ Discontinuation with Surgical intervention	1 (6.7%)	0 (0.0%)
└─ Re-initiation after thrombus progression	2 (13.3%)	0 (0.0%)

Data are presented as number (percentage) unless otherwise indicated. The subcategories under treatment discontinuation were mutually exclusive and collectively accounted for all treatment discontinuation events. SMD, standard maintenance-dose anticoagulation; LMD, low maintenance-dose anticoagulation.

### 3.3 IDDVT resolution

For the secondary outcome of IDDVT resolution, the cumulative incidence at the end of follow-up was 67.11% (95% CI 0.56–0.78) in the LMD group and 71.05% (95% CI 0.61–0.82) in the SMD group. No significant difference was observed between the CIFs (Gray's test *P* = 0.726). Consistent with this, the Fine–Gray competing risk model yielded an SHR of 0.93 (95% CI 0.64–1.36; *P* = 0.721) for the LMD group. The CIFs for both groups are presented in Figure 4.

**Figure 4:**
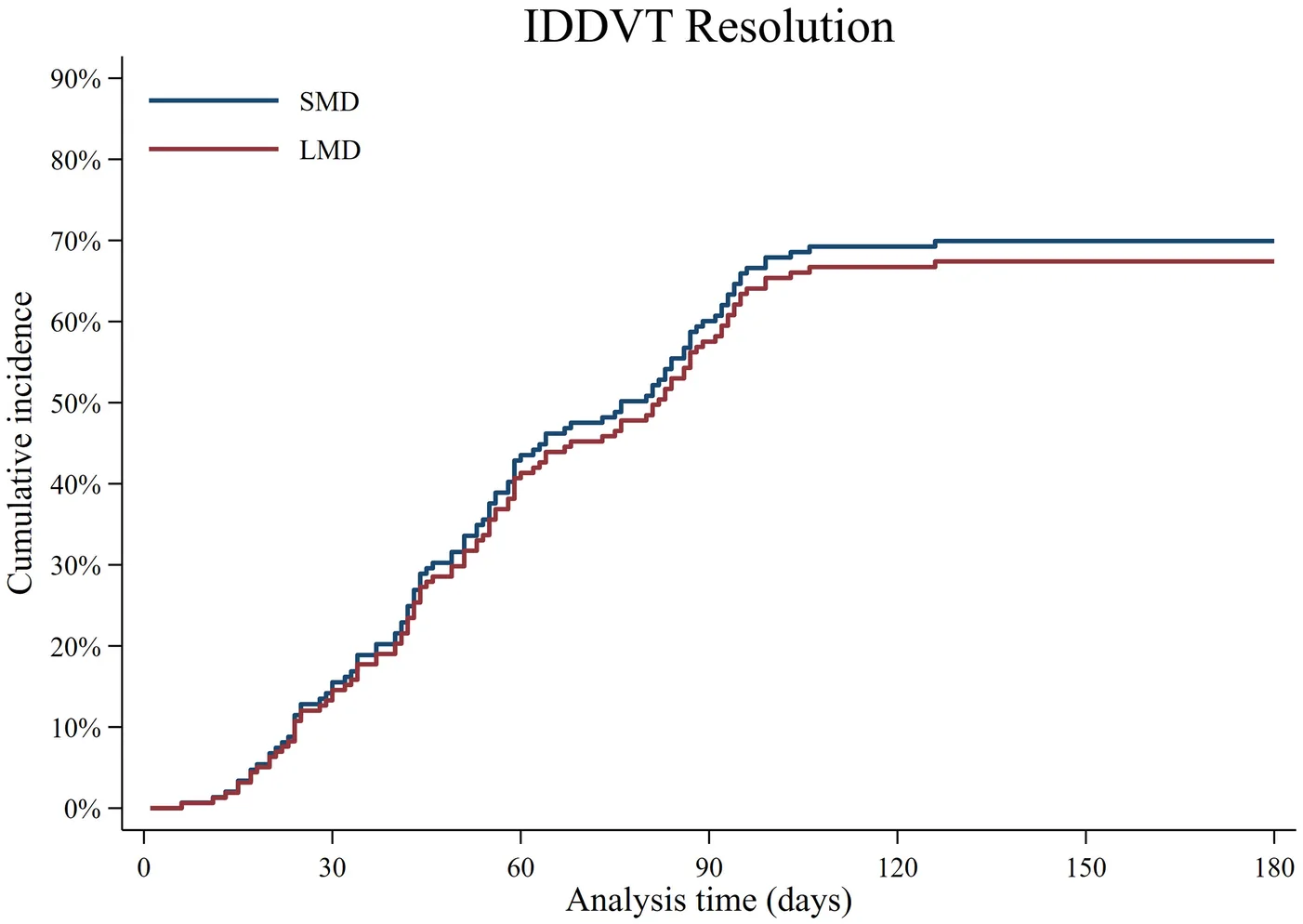
Cumulative incidence of IDDVT resolution in the SMD and LMD groups. No significant difference was observed between the two groups (SHR = 0.93; 95% CI, 0.64–1.36; *P* = 0.721).

### 3.4 Hematuria outcomes

Within the first month of treatment, dipstick positive hematuria was observed in 23 patients in the SMD group compared with 12 in the LMD group (*P* = 0.034). During the subsequent follow-up period (months 2–6), hematuria events occurred in 9 patients in the SMD group versus 7 in the LMD group (*P* = 0.597). The distribution of hematuria grades is presented in Figure 5.

**Figure 5:**
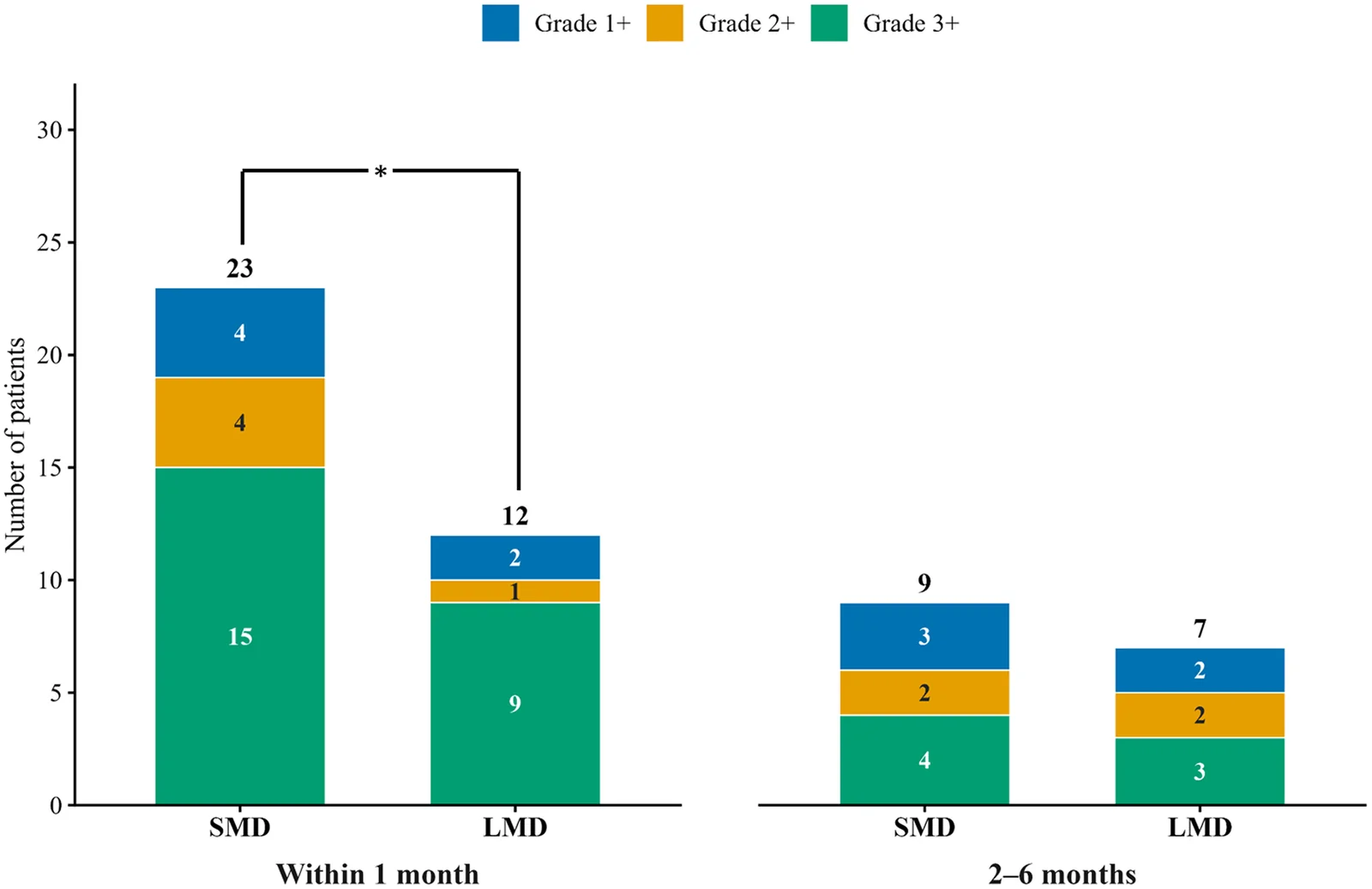
Distribution of dipstick positive hematuria grades in the SMD and LMD groups during the 1-month and 2–6-month follow-up periods. Dipstick hematuria was graded as 1+ (mild), 2+ (moderate), or 3+ (marked). The SMD group had a significantly higher rate of hematuria within the first month (*P* = 0.034).

## 4 Discussion

Current clinical guidelines generally recommend at least 3 months of anticoagulation for patients with high-risk IDDVT, with a preference for DOACs (11, 12). However, the evidence supporting these recommendations is largely extrapolated from large randomized trials focusing on PDVT or PE. These studies predominantly enrolled non-surgical populations and frequently excluded perioperative patients with a high risk of bleeding. Consequently, the optimal anticoagulation intensity and strategy for patients with specific clinical profiles, such as those in the postoperative recovery phase, remain a subject of controversy (13).

While studies such as EINSTEIN-CHOICE have investigated the use of low-dose rivaroxaban for the extended treatment of VTE (7, 14), research concerning anticoagulation dosing for acute IDDVT—particularly in patients with high bleeding risk factors—remains scarce. In our study, no statistically significant differences were detected between the de-escalated and standard-maintenance regimens in thrombus progression or complete thrombus resolution. However, the wide confidence interval for thrombus progression (SHR, 1.29; 95% CI, 0.48–3.46) indicates substantial uncertainty around the estimated treatment effect. These findings should therefore not be interpreted as evidence of equivalent antithrombotic efficacy. This finding aligns with the results reported by Fan et al. (15), which suggested that low-dose anticoagulation may be a potential strategy for reducing thrombus propagation without substantially increasing bleeding risk in critically ill patients with multiple comorbidities. However, the differences in patient populations and study settings limit direct comparison. A study by Li et al. (13) of patients with IDDVT following thoracic surgery also reported no statistically significant difference in thrombus progression between different anticoagulation dosing strategies. Taken together, these findings provide indirect support for further evaluation of de-escalated anticoagulation, although differences in study populations and study designs should be considered when interpreting the available evidence.

Wang et al. (16) observed a significantly increased risk of CRNMB in the standard-dose group (HR 2.28). This bleeding signal is highly consistent with our observations: the incidence of CRNMB was significantly higher in the standard-maintenance group, primarily occurring in the early postoperative period. This finding has important clinical implications. Surgical patients, particularly those undergoing procedures on organs with rich blood supply, remain in a high-risk window for bleeding due to incomplete wound healing. In this context, even bleeding events not classified as major bleeding (e.g., macroscopic hematuria) may lead to anticoagulation interruption or dose adjustment, thereby complicating anticoagulation management (17). Therefore, careful consideration of both thrombotic and bleeding risks is particularly important during the early postoperative period.

Bleeding risk during anticoagulation is heterogeneous and may not be fully captured by conventional clinical variables alone. Recent evidence from the RIETE registry showed that low LDL-C levels were associated with an increased risk of bleeding in patients with VTE receiving anticoagulant therapy, suggesting that laboratory-based factors may provide additional information for individualized bleeding-risk assessment (18). Although LDL-C was not systematically measured in our cohort, these findings highlight the potential value of integrating patient characteristics, surgical factors, bleeding history, and relevant laboratory parameters into individualized bleeding-risk assessment, particularly in the early postoperative period.

The differences in efficacy findings between the two studies may reflect differences in study populations and clinical settings. The study by Wang et al. included a substantial proportion of patients with persistent risk factors, such as active cancer, whereas thrombosis in our cohort was predominantly associated with transient provoking factors, such as surgical trauma and immobilization. Our cohort also included an older patient population. Notably, the subgroup analysis of patients aged >65 years did not demonstrate a statistically significant difference in VTE recurrence between dosing regimens.

The absence of a statistically significant difference in thrombotic outcomes between the two dosing regimens may be compatible with the distinct pathophysiological features of IDDVT. Compared to proximal DVT, IDDVT is typically associated with a smaller thrombus burden and a relatively lower rate of proximal extension and recurrence during its natural history (12). The prognostic significance of venous thrombosis may also depend on the distribution and extent of venous involvement. In the RIETE registry, concomitant ipsilateral superficial vein thrombosis in patients with lower-limb DVT was associated with a higher risk of subsequent PE, but not recurrent DVT (19). These findings highlight the heterogeneity of thrombotic phenotypes and the need to consider anatomical involvement when assessing thrombotic risk. Meanwhile, patients with cancer-associated IDDVT generally face higher risks of recurrence and bleeding (20, 21). In populations where transient factors like surgery are the predominant triggers, local factors may drive thrombosis more than systemic hypercoagulability. Therefore, the optimal anticoagulation intensity in this population remains uncertain and warrants prospective evaluation.

In our analysis, we observed no statistically significant difference in thrombus resolution rates between the two dosing regimens. As emphasized by Wang et al., although the standard-dose regimen may reduce the risk of recurrence, its associated bleeding risk should not be overlooked. In the study by Li et al., the half-dose regimen was associated with a reduced need for post-discharge anticoagulant therapy, highlighting the potential pharmacoeconomic and adherence-related benefits of a de-escalation strategy. However, the absence of a statistically significant difference should not be interpreted as evidence of equivalent efficacy, particularly given the observational design and limited sample size.

This study has several limitations. First, as a single-center retrospective study with a relatively small sample size spanning over a decade, inherent selection and chronological biases cannot be entirely excluded. Although year of surgery was incorporated into the propensity score model to account for potential temporal differences, residual confounding from unmeasured or incompletely documented factors remains possible. Second, patients with a creatinine clearance (CrCl) <50 mL/min were excluded, which limits the generalizability of our findings, particularly to older postoperative patients with moderate-to-severe renal impairment. Renal dysfunction may affect rivaroxaban exposure, dose selection, and bleeding risk; therefore, the safety and effectiveness of dose de-escalation in this population remain uncertain. Third, rivaroxaban dose was selected in routine practice rather than randomly assigned. Treating physicians may have preferentially prescribed a low- or standard-dose regimen according to patients’ postoperative condition and perceived bleeding risk, potentially introducing confounding by indication. Although propensity score matching balanced the measured covariates included in the model, it could not eliminate residual confounding related to clinical judgment, operative complexity, postoperative status, or other unmeasured or incompletely documented factors. In addition, the timing of follow-up ultrasonography was not strictly standardized, which may have introduced potential surveillance bias in detecting thrombus progression or complete resolution.

## 5 Conclusion

In this single-center retrospective cohort of patients who developed isolated distal deep vein thrombosis after urological surgery, treatment with low-dose rivaroxaban (10 mg once daily) was associated with a lower incidence of clinically relevant non-major bleeding than treatment with 20 mg once daily. No statistically significant differences were detected between the two regimens in thrombus progression or complete thrombus resolution. However, these findings should not be interpreted as demonstrating equivalent antithrombotic efficacy, given the nonrandomized treatment allocation, potential residual confounding, limited sample size, and wide confidence intervals for thrombotic outcomes. The results are therefore hypothesis-generating and support prospective, adequately powered studies—preferably randomized non-inferiority trials—to determine whether dose de-escalation can provide an acceptable balance between thrombotic protection and bleeding risk in selected postoperative patients with isolated distal deep vein thrombosis.

## Data Availability

The raw data supporting the conclusions of this article will be made available by the authors, without undue reservation.

## References

[1] Mazzolai L Ageno W Alatri A Bauersachs R Becattini C Brodmann M. Second consensus document on diagnosis and management of acute deep vein thrombosis: updated document elaborated by the ESC working group on aorta and peripheral vascular diseases and the ESC working group on pulmonary circulation and right ventricular function. Eur J Prev Cardiol. (2022) 29(8):1248–63. 10.1093/eurjpc/zwab088 34254133

[2] Hebert KJ Matta R Horns JJ Paudel N Das R Kohler TS. Risk of postoperative thromboembolism in men undergoing urological prosthetic surgery: an assessment of 21,413 men. J Urol. (2022) 208(4):878–85. 10.1097/JU.0000000000002801 35686836

[3] Kakkos SK Gohel M Baekgaard N Bauersachs R Bellmunt-Montoya S Black SA. Editor’s choice—european society for vascular surgery (ESVS) 2021 clinical practice guidelines on the management of venous thrombosis. Eur J Vasc Endovasc Surg. (2021) 61(1):9–82. 10.1016/j.ejvs.2020.09.023 33334670

[4] Guarascio M Valeriani E Girardi L Candeloro M Pannunzio A Palumbo IM. Anticoagulant treatment for isolated distal deep vein thrombosis: a systematic review and meta-analysis[J/OL]. Haematologica. (2025) 110(10):2332–42. 10.3324/haematol.2024.286963 40400471 PMC12485350

[5] Tan M Lurie F Kim D-I Wakefield T Parsi K Davies AH. Management of isolated distal deep venous thrombosis. Phlebology. (2024) 39(2):143–6. 10.1177/02683555231211095 37908099 PMC10877996

[6] Bikdeli B Caraballo C Trujillo-Santos J Galanaud JP Di Micco P Rosa V. Clinical presentation and short- and long-term outcomes in patients with isolated distal deep vein thrombosis vs proximal deep vein thrombosis in the RIETE registry. JAMA Cardiol. (2022) 7(8):857. 10.1001/jamacardio.2022.1988 35830171 PMC9280612

[7] Weitz JI Lensing AWA Prins MH Bauersachs R Beyer-Westendorf J Bounameaux H. Rivaroxaban or aspirin for extended treatment of venous thromboembolism. N Engl J Med. (2017) 376(13):1211–22. 10.1056/NEJMoa1700518 28316279

[8] Davis NF Fanning DM McGuire BB Carroll GT Flood HD. Perioperative management of chronic anticoagulation therapy in urological patients: a cross-sectional survey of practice. Ir J Med Sci. (2011) 180(4):823–8. 10.1007/s11845-011-0738-9 21796507

[9] Huang Z Sun H Li D Cai Z Chen M Ma S. Follow-up study of isolated calf muscular vein thrombosis for anticoagulant therapy after primary hip and knee arthroplasty. Arch Orthop Trauma Surg. (2023) 144(1):7–13. 10.1007/s00402-023-05011-9 37548684

[10] Kim T-S Min B-H Baek S-Y Kim K Min Y-W Lee H. Antithrombotic therapy increases the risk of bleeding after endoscopic submucosal dissection for early gastric cancer: a propensity score-matched analysis. Cancers (Basel). (2023) 15(10):2844. 10.3390/cancers15102844 37345180 PMC10216250

[11] Ageno W Mantovani LG Haas S Kreutz R Monje D Schneider J. Patient management strategies and long-term outcomes in isolated distal deep-vein thrombosis versus proximal deep-vein thrombosis: findings from XALIA. TH Open. (2019) 03(01):e85–93. 10.1055/s-0039-1683968 PMC6524913 31249987

[12] Makedonov I Galanaud JP Kahn SR. Significance and management of isolated distal deep vein thrombosis. Curr Opin Hematol. (2021) 28(5):331–8. 10.1097/MOH.0000000000000670 34267078

[13] Li Y Ding J Shen L Yang J Wang H Zhou Y. Risk factors and anticoagulation therapy in patients with isolated distal deep vein thrombosis in the early post-operative period after thoracic surgery. Front Surg. (2021) 8:671165. 10.3389/fsurg.2021.671165 33996889 PMC8113622

[14] Mo M Fukuda I Nakamura M Yamada N Takayama M Maeda H. Effectiveness and safety of the direct oral anticoagulant in acute distal deep vein thrombosis: from the prospective multicenter observational study, j’xactly, in Japan. Phlebology. (2023) 38(1):4–15. 10.1177/02683555221141310 36433754

[15] Fan S Fu B Liu J Liu Y Wang X Chen H. Anticoagulation in critically ill patients with isolated distal deep vein thrombosis: a retrospective cohort study. BMC Cardiovasc Disord. (2025) 25(1):646. 10.1186/s12872-025-05087-8 40890596 PMC12400710

[16] Wang B Wang Q Ji Y Zhang Y Qiao T. The clinical outcomes of different doses of rivaroxaban in patients with isolated distal deep vein thrombosis. J Vasc Surg Venous Lymphat Disord. (2024) 12(2):101653. 10.1016/j.jvsv.2023.07.008 37490950 PMC11523455

[17] Riikonen J Pakarainen T Siltari A Pienimäki J-P Koskimäki J Murtola TJ. Urine colour as an indicator for anastomotic leakage after robot-assisted radical prostatectomy. Scand J Urol. (2020) 54(3):201–7. 10.1080/21681805.2020.1750474 32308088

[18] Siniscalchi C Meschi T Di Micco P Imbalzano E Hernández-Blasco L Mahé I. Low-density lipoprotein cholesterol levels and bleeding risk in venous thromboembolism. JAMA Netw. Open. (2025) 8(5):e259467. 10.1001/jamanetworkopen.2025.9467 40343701 PMC12065040

[19] Dubois-Silva Á Barbagelata-López C Piñeiro-Parga P Francisco I Falgá C Tirado R. Prognostic significance of concomitant superficial vein thrombosis in patients with deep vein thrombosis of the lower limbs. Thromb Haemostasis. (2021) 121(12):1650–9. 10.1055/a-1414-5055 33677826

[20] Brown C Brandt W Wang T-F Delluc A Carrier M. Incidence of recurrent venous thromboembolism and bleeding complications in patients with cancer and isolated distal deep vein thrombosis. Thromb Res. (2023) 228:81–4. 10.1016/j.thromres.2023.05.027 37301116

[21] Ogino Y Ishigami T Sato R Nakahashi H Minamimoto Y Kimura Y. Direct oral anticoagulant therapy for isolated distal deep vein thrombosis associated with cancer in routine clinical practice. J Clin Med, 2021, 10(20): 4648. 10.3390/jcm10204648 34682770 PMC8539627

